# Dietary Tartary Buckwheat Flavonoids Enhance Antioxidant Capacity and Regulate Lipid Metabolism via the AMPK Pathway in Liangshan Yanying Chickens

**DOI:** 10.3390/vetsci13040375

**Published:** 2026-04-13

**Authors:** Dongdong Li, Yi Zhang, Anqiang Lai, Binlong Chen, Silu Wang, Caiyun Sun, Zhiqiu Huang, Zengwen Huang

**Affiliations:** College of Animal Science, Xichang University, Xichang 615000, China; xingguangldd@foxmail.com (D.L.); xcc20220298@xcc.edu.cn (Y.Z.); laq3521@126.com (A.L.); binlong2369@163.com (B.C.); xcc04000024@xcc.edu.cn (S.W.); xcc20210212@xcc.edu.cn (C.S.); xcxy@xcc.edu.cn (Z.H.)

**Keywords:** tartary buckwheat flavonoids, Liangshan Yanying chicken, growth performance, antioxidant capacity, lipid metabolism

## Abstract

The present work was designed to investigate the impacts of tartary buckwheat flavonoids (TBF) on the growth performance and physiological metabolism of Liangshan Yanying chickens. A total of 144 healthy 4-week-old Liangshan Yanying chickens of uniform body weight were randomly divided into four groups. Each group consisted of six replicates with six chickens per replicate. The control group was fed a basal diet, whereas the treatment groups received the same basal formulation supplemented with TBF at concentrations of 20, 40, and 60 mg/kg, respectively. The entire feeding trial lasted for 10 consecutive weeks. Growth performance, serum parameters, bone quality, slaughter traits, and hepatic lipid metabolism were determined and statistically analyzed. Results showed that dietary TBF supplementation had no significant impact on the overall growth performance (*p* > 0.05); however, final body weight and average daily weight gain displayed a positive linear trend in response to increasing TBF levels (0.05 < *p* < 0.1). For serum parameters, TBF supplementation significantly increased total antioxidant capacity (*p* < 0.05) and decreased malondialdehyde content (*p* < 0.05) in a linear manner. Specifically, compared with the control group, the 60 mg/kg TBF group increased T-AOC by approximately 64.6% and reduced MDA by approximately 67.9%, demonstrating a robust antioxidant effect. A linear increase in high-density lipoprotein cholesterol levels approaching significance (*p* = 0.055) was also observed. A significant quadratic regulatory effect of TBF was observed on serum glucose concentrations (*p* < 0.05), whereby the 60 mg/kg TBF dose reduced serum glucose by 15.6% relative to the control (*p* < 0.05), reflecting a robust hypoglycemic effect. Regarding bone quality, supplementation with 20 and 60 mg/kg TBF significantly elevated tibial phosphorus content relative to the 0 mg/kg TBF group (*p* < 0.05). Regression analysis showed that increasing dietary TBF levels linearly elevated tibial calcium content (*p* < 0.05) and resulted in linear increasing trends in tibial ash content, tibial phosphorus content, femur ash content, and femur calcium content (0.05 < *p* < 0.1). Concerning slaughter performance, dietary TBF inclusion resulted in a significant linear rise in breast muscle percentage (*p* < 0.05). At the molecular level, TBF upregulated the mRNA expression of hepatic AMPKα1 and CPT1, while concurrently downregulating the expression of FAS and ACC (*p* < 0.05). Collectively, dietary TBF supplementation in Liangshan Yanying chickens effectively improved antioxidant capacity, promoted tibial calcium and phosphorus deposition, regulated hepatic fatty acid oxidation and synthesis via AMPK-related genes, and enhanced lean meat deposition, with no adverse effects on growth performance under the experimental conditions.

## Simple Summary

With antibiotic restrictions in livestock production, plant-derived bioactive additives are a research priority. Flavonoids are plant-derived bioactive components with well-documented antioxidative, anti-inflammatory, and lipid-regulatory properties. Tartary buckwheat flavonoids (TBF) are hypothesized to enhance the production efficiency and improve the health status of Liangshan Yanying chickens. In the present work, we evaluated the impacts of dietary TBF inclusion in Liangshan Yanying chickens, and our results revealed that TBF exerted no adverse impacts on growth performance, but it significantly enhanced serum antioxidant capacity, promoted tibial calcium and phosphorus deposition, increased lean meat deposition, and regulated hepatic fatty acid oxidation and synthesis via AMPK-related genes. This work supplies theoretical backing and data evidence for the application of tartary buckwheat flavonoids in Liangshan Yanying chicken production.

## 1. Introduction

Liangshan Yanying chicken is a remarkable and high-quality indigenous chicken breed in China, originally from Meigu County and Leibo County, Sichuan Province. It is locally known as “high legged chickens” due to its long legs and feet. Its breeding history can be traced back to 220 BC, and it has long inhabited the alpine mountainous areas with an altitude of 1800–2800 m and large diurnal temperature variations [1]. Compared with fast-growing broilers, it has unique advantages such as strong roughage tolerance, high stress resistance, and excellent meat quality. At present, relying on the unique ecological resources of the Liangshan Mountains, the breeding of Yanying chicken has formed an industrial pattern dominated by ecological free-range breeding and supplemented by scale demonstration, but it is still in the critical stage of transformation from traditional breeding to modern industrialization. Under the condition of intensive breeding, the Liangshan Yanying chicken industry has gradually exposed prominent problems such as degradation of breed characteristics, increased pressure of disease prevention and control, and weakened advantages of ecological quality, which have seriously restricted the improvement of industrial quality and efficiency and high-quality development. Therefore, how to scientifically improve breeding efficiency and promote the healthy development of the industry has become an important issue that urgently needs to be solved.

Against the backdrop of current development trends in the livestock and poultry breeding industry, as well as the comprehensive implementation of policies banning and restricting antimicrobial use, the development of safe, efficient, green, and eco-friendly novel bioactive additives as alternatives to antibiotics has become a research hotspot and an inevitable trend in livestock and poultry production [2]. Flavonoids, which constitute a significant class of secondary metabolites in plants, are broadly distributed in multiple plant tissues, encompassing fruits, vegetables, grains, seeds, leaves, barks, and other related tissues [3]. According to their structural differences, flavonoids can usually be divided into seven subclasses: flavones, isoflavones, flavanones, anthocyanins, flavonols, flavanols and chalcones [3,4]. Accumulating research indicates that flavonoids possess diverse biological regulatory properties, among which antioxidant activity acts as a central underlying mechanism [5,6]. By effectively scavenging free radicals and alleviating oxidative stress, flavonoids can further regulate critical inflammatory signaling cascades including NF-κB and MAPK pathways, thereby exerting notable anti-inflammatory effects [7,8]. In addition to their anti-inflammatory capacity, flavonoids also display direct antibacterial effects against Gram-negative bacteria [9]; together, these activities contribute to the maintenance of intestinal barrier function and the stability of gut microbiota, thus supporting intestinal health [10,11]. Of particular importance, the antioxidant property of flavonoids is closely associated with their regulatory role in lipid metabolism. Through attenuating lipid peroxidation and activating signaling pathways such as AMPK, flavonoids suppress lipid synthesis while accelerating lipid decomposition, thereby efficiently modulating lipid accumulation and metabolic homeostasis [12,13]. In poultry nutrition, these compounds have attracted increasing interest as promising feed additives, especially for their roles in enhancing antioxidant capacity, regulating lipid metabolism, and controlling fat deposition. However, their effects vary considerably depending on flavonoid type, dosage, and production conditions [14]. Previous studies have found that adding 20 mg/kg hawthorn leaf total flavonoids to broiler feed can enhance growth performance and breeding economic benefits of broilers, and effectively boost the nutritional and sensory quality of their meat [15]. Another study reported that dietary supplementation with 20 mg/kg quercetin-hesperidin mixture (1:4) significantly improved antioxidant capacity and decreased lipid levels in serum and pectoral muscle, while increasing the polyunsaturated fatty acid proportion in the pectoral muscle of AA broilers [16]. Akter et al. (2024) reported that adding a 0.6 g/kg plant flavonoid mixture to broiler feed can significantly promote their growth, enhance their digestive and absorption functions, increase the number of beneficial intestinal microflora and the content of beneficial serum metabolites, and thus effectively improve meat quality [17]. Studies on quail have found that dietary supplementation of garlic powder can improve the growth performance of Japanese quails and reduce lipid oxidation in their meat during storage [18]. In view of the above progress and current challenges in the intensive breeding of Liangshan Yanying chickens, flavonoid-based bioactive additives may provide an effective approach to improve productivity and lay a solid foundation for subsequent research.

Given the local resource advantages of tartary buckwheat in Liangshan, tartary buckwheat flavonoids (TBF), extracted from tartary buckwheat, may become a valuable and sustainable bioactive resource for local chicken breeding. TBF are a class of natural bioactive components, whose main components include rutin and quercetin, both of which are representative components with significant physiological activities among flavonoids. Previous studies have shown that, in mouse models, TBF can alleviate high-fat diet-induced renal fibrosis by inhibiting the MAPK and TGF-β1/Smad signaling pathways, demonstrating favorable anti-inflammatory and anti-fibrotic activities [19]. At present, research on the application of TBF in animal production is insufficient, especially in local characteristic livestock and poultry. A study in pigs demonstrated that dietary supplementation with 40 mg/kg TBF significantly improved the average daily gain of weaned piglets, elevated serum immunoglobulin levels, and enhanced the immune capacity of the body [20]. Meanwhile, TBF supplementation also showed positive effects on nutrient digestibility in weaned piglets, and its combination with Lactobacillus plantarum could exert a synergistic effect on improving the digestibility of gross energy, dry matter and phosphorus [20].

Accordingly, the present work was designed to investigate the impacts of dietary TBF on growth performance, serum biochemical indicators, bone quality, slaughter performance and liver lipid metabolism of Liangshan Yanying chickens. Findings from this research are anticipated to offer a theoretical foundation and empirical data to support the application of TBF in the breeding of local chicken breeds, and also promote the industrialization development of Liangshan Yanying chickens.

## 2. Materials and Methods

### 2.1. Experimental Design and Animal Management

The present study was carried out at the Greenhouse Experimental Base of Xichang University in Sichuan Province, China. The animal experiments were approved by the Animal Ethics Committee of the Animal Science College of Xichang University (Approval Number: 2024016) and were carried out in accordance with the prescribed requirements.

A total of 144 healthy 4-week-old Liangshan Yanying chickens with similar body weight were randomly divided into four experimental groups at the replicate level following a completely randomized design, where each group consisted of 6 replicates and each replicate contained 6 chickens. Birds in each replicate were kept in separate cages (90 cm × 60 cm × 50 cm, L × W × H), with a stocking density of 900 cm^2^/bird. All cages were evenly arranged in the broiler house to minimize environmental biases. The basal diet formula is shown in Table 1. Dietary TBF was supplemented at 20, 40, and 60 mg/kg in the treatment groups, respectively. The feeding trial spanned 10 weeks (from 4 to 14 weeks of age), and all diets were provided as mash ad libitum. The basal diet was formulated according to the Chinese Feeding Standard for Chickens [21]. The TBF (purity 50%) was supplied by Fufeng Snoot Biotechnology Co., LTD (Fufeng, Shaanxi, China). During the entire experimental period, environmental conditions in the broiler house were strictly controlled to ensure stability: the ambient temperature in the indoor ranged from 22 to 25 °C, with relative humidity between 55% and 65%, and a photoperiod of 18 h light:6 h dark was implemented to satisfy the physiological requirements of the growing chickens. Continuous mechanical ventilation was implemented to keep the indoor air fresh and maintain normal concentrations of harmful gases within the standard range.

### 2.2. Growth Performance

Individual body weight (BW) of each chicken was measured at 4 weeks of age (initial BW) and 14 weeks of age (final BW) using an electronic balance. The average daily gain (ADG) throughout the 10-week feeding trial (from 4 to 14 weeks of age) was calculated as (final BW minus initial BW) divided by the total number of days in the trial. During the experiment, accurately record the amount of feed added and calculate the average daily feed intake (ADFI). Feed conversion ratio (FCR), an indicator of feed utilization efficiency, was determined as the ratio of ADFI to ADG.

### 2.3. Serum Biochemical Indicators

At the end of the 10-week feeding trial (14 weeks of age), one chicken was randomly chosen from each replicate for serum collection, resulting in 6 chickens per treatment group. Blood samples of 5 mL per individual were harvested from the brachial vein via sterile disposable syringes. After the blood sample was centrifuged at 3000 r/min for 15 min, the upper serum layer was separated and placed in a sterile EP tube, which was then stored at −80 °C for testing. Serum levels of albumin (ALB), total cholesterol (TC), low-density lipoprotein cholesterol (LDL-C), triglyceride (TG), high-density lipoprotein cholesterol (HDL-C), creatinine, urea, glucose, and total protein (TP) were measured with matched commercial assay kits obtained from Nanjing Jiancheng Bioengineering Institute (Nanjing, China). All assay kits were rigorously validated by the manufacturer, with intra-assay coefficients of variation (CV) < 5% and inter-assay CV < 10% for all measured indicators, which meets the international quality standards for clinical biochemical testing. All samples were assayed in triplicate, and all procedures were carried out strictly in accordance with the manufacturer’s protocols.

### 2.4. Serum Antioxidant and Immune Function Indicators

The immune function was evaluated via the detection of key humoral factors, including immunoglobulins (IgA, IgG, IgM) and the pro-inflammatory cytokines tumor necrosis factor-α (TNF-α) and interleukin-2 (IL-2). For the estimation of antioxidant status, the measured indices included malondialdehyde (MDA), catalase (CAT), total antioxidant capacity (T-AOC), glutathione peroxidase (GSH-Px), and superoxide dismutase (SOD). Quantification of these parameters was performed with commercial ELISA kits obtained from Shanghai Enzyme-linked Biotechnology Co., Ltd. (Shanghai, China). All ELISA kits were validated by the manufacturer, with intra-assay coefficients of variation < 8% and inter-assay coefficients of variation < 12% for all target analytes, ensuring the reliability and sensitivity of the detection. Each sample was analyzed in triplicate during testing, and all handling steps were performed strictly in accordance with the corresponding kit protocols. Absorbance signals were recorded at the appropriate wavelengths using a microplate reader (Waltham, MA, USA).

### 2.5. Bone Indicators

After blood collection, the selected chickens were humanely euthanized by severing the trachea at the cervical vertebrae. Subsequently, the tibia and femur of the right leg were rapidly dissected, and all remaining muscle and connective tissue were carefully trimmed off. The weights of the tibia and femur were measured using an electronic balance, and their length was determined using a vernier caliper. The diameter of the midshaft of both bones was also measured using a vernier caliper. The formulas for tibia plumpness and femur plumpness were calculated as follows: (midshaft diameter of bone × 3.14/bone length) × 100%. Bone strength was assessed using an Instron 5565 universal testing machine (Instron Corporation, Norwood, MA, USA) with a crosshead speed of 5 mm/min. After determining the bone strength, the bone was dried in a 65 °C oven until a constant weight was achieved, and then it was burned in a muffle furnace at 550 °C for 6 h. The bone ash content was calculated as (ash weight/dry bone weight) × 100%. The calcium and phosphorus contents in the bone ash were determined by ethylenediaminetetraacetic acid (EDTA) titration and ammonium molybdate-vanadate spectrophotometry, respectively.

### 2.6. Expression of Genes Related to Liver Lipid Metabolism

Following euthanasia, liver tissue samples were rapidly collected, snap-frozen in liquid nitrogen, and stored at −80 °C for total RNA extraction. Total RNA was extracted using Trizol reagent (Sangon Biotech, Shanghai, China)according to the instructions, and RNA purity and concentration were determined using a NanoDrop 2000 spectrophotometer (Thermo Fisher Scientific, Waltham, MA, USA). Complementary DNA was synthesized from 1 μg of total RNA using a reverse transcription kit. The transcript levels of genes were detected in this study, including acetyl-CoA carboxylase (ACC), carbohydrate response element-binding protein 1 (ChREBP1), fatty acid-binding protein (FABP), fatty acid synthase (FAS), glycerol-3-phosphate acyltransferase 1 (GPAT1), sterol regulatory element-binding protein-1C (SREBP-1C), peroxisome proliferator-activated receptor α (PPARα), AMP-activated protein kinase α1 (AMPKα1), and carnitine palmitoyltransferase 1 (CPT1). All the above genes were analyzed by quantitative real-time PCR (qRT-PCR) on a real-time PCR system using SYBR Green PCR Master Mix (Vazyme Biotech, Nanjing, China), with β-actin as the internal reference. Gene expression levels were normalized to β-actin, and the relative expression was calculated using the 2^−ΔΔCt^ method according to established protocols for qRT-PCR data normalization. The primer sequences used for qRT-PCR are listed in Table 2.

### 2.7. Slaughter Performance Indicators

One chicken was randomly selected from each replicate and euthanized individually for the determination of slaughter performance, separately from those used for sample collection. Live weight was measured first, and then the chicken was processed according to standard slaughter procedures, including exsanguination, defeathering, and evisceration. Dressed weight, half-eviscerated weight, and eviscerated weight were recorded separately. Subsequently, the thigh muscle and breast muscle were completely separated, weighed, and recorded. The slaughter performance-related indices were calculated. The formulas are as follows:

Dressed percentage = (Dressed weight/Live weight) × 100%

Half-eviscerated percentage = (Half-eviscerated weight/Live weight) × 100%

Eviscerated percentage = (Eviscerated weight/Live weight) × 100%

Breast muscle percentage = (Breast muscle weight/Eviscerated weight) × 100%

Thigh muscle percentage = (Thigh muscle weight/Eviscerated weight) × 100%

### 2.8. Statistical Analysis

All statistical analyses were performed using SAS 9.4 software (SAS Institute Inc., Cary, NC, USA). Normality and homogeneity of variances were tested for all variables. Data that violated these assumptions were transformed before analysis. The general linear model (GLM) procedure was applied to analyze the effects of different treatments. In this model, dietary treatment was regarded as the fixed effect, and replicate was treated as the random effect. Least squares means were calculated for each treatment group, and multiple comparisons were performed with Duncan’s test. Polynomial contrasts were applied to assess linear and quadratic effects of increasing TBF supplementation. A probability value of *p* < 0.05 was defined as statistical significance, and 0.05 ≤ *p* < 0.10 was considered to indicate a tendency toward significance. Results are presented as means with standard error of the mean (SEM), and exact *p*-values are provided for all statistical comparisons.

## 3. Results

### 3.1. Impact of TBF on the Growth Performance of Liangshan Yanying Chickens

As shown in Table 3, no significant differences were observed in body weight (BW) at the start of the trial (4 weeks of age) among all treatment groups. Results from the analysis of variance (ANOVA) indicated that dietary supplementation with TBF had no significant effect on final body weight (BW at 14 wk), body weight gain (BWG, 4–14 wk), feed conversion ratio (FCR, 4–14 wk), or average daily feed intake (ADFI, 4–14 wk) of Liangshan Yanying chickens (*p* > 0.05). However, polynomial contrast analysis revealed a linear increasing trend for BW at 14 wk (*p* = 0.083) and BWG (4–14 wk) (*p* = 0.075) in response to increasing TBF levels, while no significant differences were found in other growth performance indices (*p* > 0.05).

### 3.2. Impact of TBF on the Serum Biochemical Indices of Liangshan Yanying Chickens

According to Table 4, dietary supplementation with TBF showed a tendency to increase serum HDL-C levels (*p* = 0.058), and this increase exhibited a linear dose-dependent relationship (*p* = 0.055). Additionally, TBF had a significant quadratic effect on serum glucose levels (*p* = 0.019), with glucose concentrations decreasing from the control to the 60 mg/kg TBF group after an initial increase at 40 mg/kg. However, no significant differences in serum ALB, TC, LDL-C, TG, Creatinine, Urea, or TP levels were observed in Liangshan Yanying chickens fed diets supplemented with TBF (*p* > 0.05).

### 3.3. Impact of TBF on Serum Immune Factors and Antioxidant Indices in Liangshan Yanying Chickens

As shown in Table 5, dietary TBF supplementation did not significantly affect serum concentrations of immunoglobulins (IgA, IgG, IgM), IL-2, or TNF-α in Liangshan Yanying chickens (*p* > 0.05). For antioxidant indices, TBF supplementation induced a significant linear increase in T-AOC (*p* = 0.018), while it significantly reduced MDA levels in a linear manner (*p* < 0.001), with the lowest MDA content observed in the 60 mg/kg TBF group. No statistically significant variations were observed in the activities of SOD, GSH-Px, or CAT among all treatment groups (*p* > 0.05).

### 3.4. Impact of TBF on the Bone Development and Bone Quality of Liangshan Yanying Chickens

As shown in Table 6, TBF supplementation led to a significant linear increase in tibia calcium content (*p* = 0.049). With increasing dietary supplementation of TBF, a linear increasing trend was also observed in tibia ash content, tibia phosphorus content, femur ash content, and femur calcium content. However, dietary supplementation with TBF did not significantly affect tibia or femur weight, length, diameter, plumpness, or strength in Liangshan Yanying chickens (*p* > 0.05).

### 3.5. Impact of TBF on the mRNA Expression of Lipid Metabolism-Associated Genes in the Liver of Liangshan Yanying Chickens

**Figure 1:**
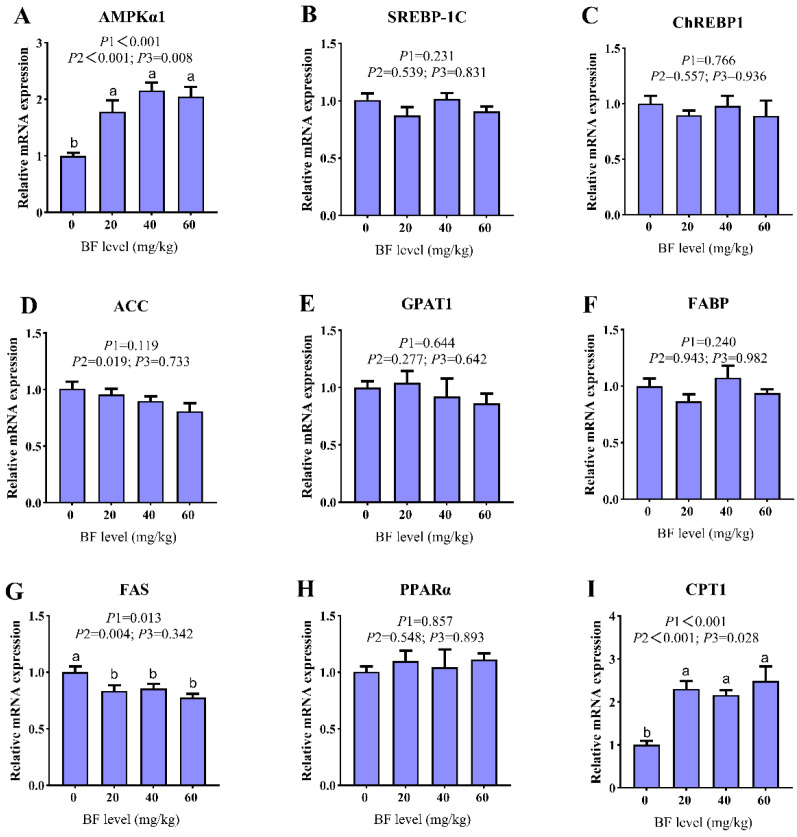
Impact of TBF on Lipid metabolism-related gene expression in the liver of Liangshan Yanying Chickens. (**A**) AMPKα1; (**B**) SREBP-1C; (**C**) ChREBP1; (**D**) ACC; (**E**) GPAT1; (**F**) FABP; (**G**) FAS; (**H**) PPARα; (**I**) CPT1. Abbreviations: AMPKα1 = AMP-activated protein kinase α1; SREBP-1C = sterol regulatory element-binding protein-1C; ChREBP1 = carbohydrate response element-binding protein 1; ACC = acetyl-CoA carboxylase; GPAT1 = glycerol-3-phosphate acyltransferase 1; FABP =fatty acid-binding protein; FAS = fatty acid synthase; PPARα = peroxisome proliferator-activated receptor α; CPT1 = carnitine palmitoyltransferase 1. *p*1, *p*2 and *p*3 represent the *p* values for ANOVA, linear and quadratic analysis, respectively. ^a,b^ Means in the same row with no common letter differ significantly (*p* < 0.05).

As depicted in Figure 1, the expression of AMPKα1 and CPT1 was significantly upregulated (ANOVA, linear, quadratic, *p* < 0.05), while the expression of FAS was significantly downregulated (ANOVA, linear, *p* < 0.05) in a linear manner with increasing TBF levels. The expression of ACC showed a significant linear decrease (*p* = 0.019), although the overall ANOVA effect was not significant (*p* > 0.05). In contrast, dietary TBF inclusion had no significant effects on the mRNA expression of SREBP-1C, ChREBP1, GPAT1, FABP, or PPARα (*p* > 0.05).

### 3.6. Impact of TBF on Carcass Traits of Liangshan Yanying Chickens

As shown in Table 7, TBF supplementation significantly increased breast muscle percentage (ANOVA, linear, *p* < 0.05), with values rising from 18.2% in the control group to 19.7% in the 60 mg/kg TBF group. However, no significant differences were observed in the other carcass performance indices of Liangshan Yanying chickens fed TBF-supplemented diets (*p* > 0.05).

## 4. Discussion

Overall, in this experiment, dietary supplementation with TBF exerted no statistically significant effects on growth performance of Liangshan Yanying chickens, including final body weight, body weight gain, ADFI, and FCR, although numerical positive trends in BW and BWG were observed. Notably, These positive trends in body weight and weight gain indicate that TBF may improve growth rate and final body weight, thereby potentially enhancing production efficiency and economic benefits in commercial poultry production. Similar observations have been reported in broiler studies. Previous studies have demonstrated that dietary supplementation with flavonoids quadratically increased average daily gain during the finishing phase, and linearly and quadratically decreased FCR in broilers, with no impact on feed intake [22]. However, not all studies show such pronounced growth-promoting effects. Paredes-Lopez et al. (2025) found that dietary supplementation with Piper aduncum flavonoids at 17.5 and 35.0 ppm maintained broiler weight gain and FCR, while reducing feed intake, without impairing overall growth performance [23]. Such discrepancies among studies may be attributed to differences in flavonoid source and dosage, poultry breed, rearing conditions, and experimental duration. Nevertheless, available evidence indicates that flavonoid supplementation generally does not negatively affect growth performance in poultry. Given that the observed trends were not statistically significant, the potential of TBF to improve growth performance should be regarded as preliminary and requires further verification.

Serum biochemical parameters are often used as an important basis for judging the health status of animals. We found that TBF supplementation linearly tended to increase HDL-C concentration, a change indicative of improved lipoprotein metabolism and reduced risk of metabolic disorders. Similar results were observed in a mouse study by Li et al. (2022), who reported that tartary buckwheat supplementation dose-dependently elevated serum HDL-C levels in mice fed a high-fat diet, while simultaneously reducing the concentrations of total cholesterol, triglycerides and low-density lipoprotein cholesterol, thereby effectively alleviating diet-induced dyslipidemia and improving overall lipid metabolic homeostasis [24]. This aligns with the lipid-regulatory potential of flavonoids—Tan et al. (2021) confirmed that quercetin significantly lowered plasma triglycerides and LDL cholesterol in obese mice, suggesting TBF’s effects may be mediated by its flavonoid constituents [25]. In addition, our study revealed that serum glucose levels exhibited a quadratic response to increasing dietary TBF, with an initial increase followed by a subsequent decrease. This glucose-modulating effect is consistent with Ma et al. (2025)’s finding that flavonoids regulate glucose carbon flux by adjusting pyruvate metabolism balance, balancing glucose utilization and lipid synthesis [26].

The antioxidant activity of plant flavonoids has been well-documented [4]. Shi et al. (2022) reported that Artemisia ordosica total flavonoids alleviated LPS-induced oxidative stress in broilers by lowering MDA production and elevating T-AOC and antioxidant enzyme (SOD, CAT, GSH-Px) activities, which was mediated through regulating the Keap1/Nrf2 signaling pathway [27]. Consistently, similar antioxidant effects of plant flavonoids were observed by Yuan et al. (2025), who found that bamboo leaf flavonoids improved hepatic antioxidant status in heat-stressed broilers via activating the Keap1-Nrf2 pathway to upregulate antioxidant gene expression [28]. Notably, the majority of previous studies have focused on the protective roles of flavonoids under stressed conditions, such as LPS challenge or heat stress, while evidence supporting their antioxidant effects under normal physiological conditions remains limited. In contrast, the present study demonstrated that TBF supplementation effectively improved antioxidant status by reducing MDA and increasing T-AOC in Liangshan Yanying chickens. This observation is strongly supported by Goliomytis et al. (2014), who confirmed that dietary quercetin also enhanced meat oxidative stability in broilers under normal rearing circumstances [29], further validating that flavonoids exert basal antioxidant effects beyond stress alleviation. In agreement with our findings, kudzu-leaf flavonoids have been shown to reduce serum MDA and improve antioxidant capacity in Yellow-feathered chickens [30]. Consistently, a recent investigation revealed that flavonoid extract from Galega orientalis Lam. improved the antioxidant capacity of broiler meat by augmenting the activities of SOD and CAT, alongside a concurrent reduction in MDA concentrations, an effect associated with modulated gut microbiota composition [31]. Collectively, the present results confirm that TBF can improve the antioxidant capacity of Liangshan Yanying chickens.

Liangshan Yanying chickens, locally known as “high-legged chickens”, frequently suffer from leg disorders during the rearing process, which is closely associated with bone quality. The present study demonstrated that TBF could increase the contents of bone ash, calcium and phosphorus to a certain extent. Although the magnitude of these changes was moderate, such improvements in bone mineralization are physiologically relevant for local high-legged chickens, as even modest increases in bone mineral deposition can effectively enhance bone mechanical strength, reduce the incidence of leg weakness, and improve walking ability and survival under intensive rearing conditions, which is of clear practical importance for animal welfare and production stability. This finding is consistent with the bone-protective effects of flavonoid-based dietary supplements reported in avian species, as Galega orientalis Lam. flavonoid extract was shown to elevate tibial Ca and P contents in broilers by promoting bone mineral deposition and improving bone tissue microstructure [32]. Similarly, total flavonoids from Rhizoma Drynariae enhanced femur and tibia bone mineral density in aged caged laying hens, accompanied by improved cortical and trabecular bone histomorphology via regulation of osteoblast and osteoclast activity [33]. Total flavonoids from Rhizoma Drynariae exert bone-protective effects by maintaining bone metabolic homeostasis. They can upregulate bone formation markers (OC, OPG, BMP2) and downregulate bone resorption markers (TRACP, CTX-1), as well as improve bone microstructure by increasing BV/TV, Tb.Th, and Tb.N while decreasing Tb.Sp [34]. In addition, total flavonoids from Rhizoma Drynariae can promote the formation of bone type H vessels via the PDGF-BB/PDGFR-β axis to enhance angiogenic-osteogenic coupling [35], and sufficient bone vascularization is a key prerequisite for nutrient supply and bone mineral deposition. These results suggest that flavonoid-based supplements such as TBF may improve bone quality in poultry through similar mechanisms, thus potentially alleviating leg disorders associated with poor bone quality in Liangshan Yanying chickens.

Consistent with the comprehensive review by Tan et al. (2022), dietary flavonoids have been widely recognized to modulate lipid metabolism and fat accretion in poultry through coordinated regulation of adipogenesis, lipolysis, and hepatic metabolic pathways [14]. The present study confirmed that TBF significantly upregulated the mRNA expression of hepatic AMPKα1 and CPT1, while concurrently downregulating FAS gene expression. This suggests that TBF can improve hepatic lipid metabolism disorders and reduce intrahepatic lipid deposition in Liangshan Yanying chickens by activating the AMPK signaling pathway, thereby promoting hepatic fatty acid oxidation and decomposition, and inhibiting de novo fatty acid synthesis. This regulatory pattern is consistent with the action of genistein, which activates AMPK signaling to enhance CPT1-mediated fatty acid β-oxidation and suppress FAS-dependent de novo lipogenesis in avian hepatic tissue [36]. Similarly, the present regulatory mechanism is highly consistent with the observation that rutin, another typical plant flavonoid, ameliorates lipid metabolism dysfunction in diabetic nonalcoholic fatty liver disease (NAFLD) by targeting the AMPK/SREBP1 signaling pathway [37], further supporting the notion that flavonoids commonly modulate hepatic lipid homeostasis through conserved AMPK-related signaling networks. In line with these findings, dietary hawthorn-leaf flavonoids have also been shown to significantly decrease serum TG, TC and LDL-C concentrations, upregulate hepatic apolipoprotein B (ApoB) and apolipoprotein VLDL I (ApoV1) mRNA expression, and alleviate hepatic lipid droplet accumulation and inflammatory cell infiltration in aged breeder hens [38]. Although these gene expression results support the involvement of the AMPK signaling pathway in TBF-mediated lipid regulation, further studies targeting protein phosphorylation and pathway inhibition are warranted to verify the exact functional mechanism.

For carcass characteristics, TBF linearly increased breast muscle percentage without influencing dressing percentage, eviscerated yield, or thigh muscle proportion, indicating a preferential enhancement of lean meat deposition. This targeted promotion of breast muscle accretion aligns with the meta-analytical evidence that dietary flavonoids can selectively modulate skeletal muscle growth in broilers without altering overall slaughter traits [22]. Some studies have also found bamboo leaf flavonoids can improve muscle fiber integrity and reduce intramuscular fat deposition, thereby facilitating the production of lean meat [39]. The underlying mechanism may be linked to flavonoid-mediated regulation of muscle protein metabolism and antioxidant status, as demonstrated by increased superoxide dismutase activity and reduced MDA levels in broiler muscle tissue following flavonoid supplementation [39,40]. This mechanistic framework is supported by studies on other flavonoid-rich botanicals. For instance, supplementation with garden cress seed powder, a natural source of bioactive flavonoids and polyphenols, has been shown to significantly increase breast muscle yield while reducing abdominal fat percentage in broilers, without adversely affecting overall dressing percentage [41].

From a practical standpoint of poultry nutrition and production, the results of this investigation support the potential use of TBF as a feed supplement for Liangshan Yanying chickens under controlled experimental conditions, offering a sustainable nutritional strategy. These findings are specific to Liangshan Yanying chickens, and further research in other chicken breeds and practical production environments is needed to verify their generalizability. Meanwhile, the present study is also in line with the global trend of reducing synthetic additives and promoting antibiotic-free feeding. Further studies should optimize its dietary inclusion level and evaluate long-term field effects, which will contribute to its practical application in commercial poultry production.

## 5. Conclusions

Dietary supplementation with TBF exerts favorable effects on Liangshan Yanying chickens, as evidenced by improved antioxidant status, enhanced bone mineralization, modulated hepatic lipid metabolism via the regulation of key genes, and increased breast muscle percentage. The findings of the present study demonstrate that TBF can serve as an eco-friendly feed supplement for Liangshan Yanying chickens, while providing empirical evidence for their application in other poultry species.

## Figures and Tables

**Table 1:** Composition of the basal diet (air-dry basis).

Ingredients	Content (%)
Maize	61.65
Soybean meal	27.4
Wheat bran	2.18
Soya oil	5.75
Limestone	1.08
Dicalcium phosphate	1.2
Choline Hydrochloride	0.1
Sodium chloride	0.3
DL-Methionine	0.11
Mineral premix ^1^	0.2
Vitamin premix ^2^	0.03
Total	100
Nutrient level (calculated values)	
Metabolizable energy (kcal/kg)	3100
Crude protein	17
Calcium	0.8
Non-Phytate P	0.35
Lysine	0.88
Methionine	0.37

^1^ Provided per kilogram of diet: Cu (CuSO_4_·5H_2_O) 8 mg, Fe (FeSO_4_·H_2_O) 80 mg, Mn (MnSO_4_·H_2_O) 80 mg, Zn (ZnSO_4_·H_2_O) 80 mg, I (KI) 0.7 mg, Se (Na_2_SeO_3_) 0.3 mg. ^2^ Provided per kilogram of diet: 2700 IU vitamin A; 400 IU vitamin D3; 10 IU vitamin E; 0.5 mg vitamin K3; 2 mg vitamin B1; 5 mg vitamin B2; 3 mg vitamin B6; 7 µg vitamin B12; 10 mg D-pantothenate; 30 mg niacin acid; 0.5 mg folic acid; 100 µg biotin.

**Table 2:** Primer sequences for the target genes.

Gene	Forward Primer	Reverse Primer
β-actin	ATCCGGACCCTCCATTGTC	AGCCATGCCAATCTCGTCTT
ACC	ATTATGTCCTGGATAACCTGGTCAAC	TCAGTGTCTTCATTAGTCGCTCAAC
ChREBP1	GATGAGCACCGCAAACCAGAGG	TCGGAGCCGCTTCTTGTAGTAGG
FABP	GAGGCTGACATTACTACTATGGATGG	TTCATTTCCCTTAACTTCTTGCTCATG
FAS	GCATAAATGATACAGAAGTTCCCACAC	CTCTCCACAGGTAATTTCTCGCTTAG
GPAT1	CAGAGGTCAAATAGAAATGGTCAAAGC	GAATGTAAGGAGCAGGTAATCAATGTG
SREBP-1C	CAGCAACAGCAGCAGTGACTC	GGTGAGGGCGGTGGGTTC
PPARα	ACGGAGTTCCAATCGC	AACCCTTACAACCTTCACAA
AMPKα1	CAACTACCTGGCTTCCGAGA	CGAAGTCATCCCGATTATAGCTC
CPT1	GGAGAACCCAAGTGAAAGTAATGAA	TGGAAACGACATAAAGGCAGAA

**Table 3:** Growth performance of Liangshan Yanying chickens fed diets supplemented with different levels of TBF.

Items	TBF, mg/kg				SEM	*p*-Value		
	0	20	40	60		ANOVA	Linear	Quadratic
BW (4 wk), g	378.3	376.2	376.7	377.5	4.9	0.990	0.928	0.763
BW (14 wk), g	1335.0	1351.2	1346.2	1382.5	16.8	0.255	0.083	0.556
BWG (4–14 wk), g	956.7	975.0	969.5	1005.0	16.6	0.240	0.075	0.611
FCR (4–14 wk)	4.675	4.505	4.578	4.545	0.1	0.671	0.488	0.503
ADFI (4–14 wk), g	63.8	62.7	63.3	65.2	1.2	0.542	0.407	0.238

Abbreviations: TBF = Tartary Buckwheat Flavonoids; BW = Body Weight; BWG = Body weight gain; FCR = Feed conversion ratio; ADFI = Average daily feed intake.

**Table 4:** Dose-dependent effects of TBF on serum biochemical parameters in Liangshan Yanying chickens.

Items	TBF, mg/kg				SEM	*p*-Value		
	0	20	40	60		ANOVA	Linear	Quadratic
ALB, g/L	14.68	14.35	13.6	14.2	0.61	0.654	0.430	0.454
TC, mmol/L	3.31	2.68	2.65	2.86	0.33	0.478	0.355	0.217
LDL-C, mmol/L	1.7	0.93	0.96	1.09	0.26	0.170	0.148	0.103
TG, mmol/L	0.58	0.58	0.55	0.44	0.05	0.245	0.080	0.311
HDL-C, mmol/L	1.4	1.71	1.53	1.75	0.10	0.058	0.055	0.637
Creatinine, μmol/L	4.67	3.45	4.52	3.73	0.64	0.476	0.550	0.737
Urea, mmol/L	0.52	0.55	0.50	0.47	0.03	0.228	0.122	0.228
Glucose, mmol/L	8.25	10.18	10.7	8.89	0.73	0.099	0.467	0.019
TP, g/L	55	46.9	48.5	46.6	3.4	0.280	0.131	0.367

Abbreviations: TBF = Tartary Buckwheat Flavonoids; ALB = Albumin; TC = Total cholesterol; LDL-C = Low-Density lipoprotein cholesterol; TG = Triglycerides; HDL-C = High-Density lipoprotein cholesterol; TP = Total protein.

**Table 5:** Dose-dependent effects of TBF on serum immune factors and antioxidant indices in Liangshan Yanying chickens.

Items	TBF, mg/kg				SEM	*p*-Value		
	0	20	40	60		ANOVA	Linear	Quadratic
immune factors								
IgA, μg/mL	44.4	44.9	47	43.6	6.7	0.986	0.995	0.767
IgG, μg/mL	271.6	316.4	385.50	319.3	41.2	0.303	0.263	0.193
IgM, μg/mL	120.1	125.2	109.7	115.9	16.7	0.926	0.712	0.972
IL-2, pg/mL	56.6	54.4	59.6	47.8	8.9	0.817	0.606	0.597
TNF-α, pg/mL	10.6	9.4	9.4	8.5	1.1	0.602	0.202	0.886
antioxidant indices								
T-AOC, U/mL	1.3	1.67	2.39	2.14	0.28	0.055	0.018	0.284
SOD, U/mL	391.9	395.7	380.5	365.2	38.9	0.945	0.591	0.810
GSH-Px, U/L	1007.7	1047.5	913.8	895.03	137.9	0.837	0.453	0.834
CAT, U/mL	1.54	2.04	2.02	1.88	0.35	0.726	0.524	0.372
MDA, nmol/mL	2.93 ^a^	1.89 ^ab^	1.25 ^b^	0.94 ^b^	0.36	0.005	<0.001	0.332

Abbreviations: TBF = Tartary Buckwheat Flavonoids; IgA = Immunoglobulin A; IgG = Immunoglobulin G; IgM = Immunoglobulin M; IL-2 = Interleukin-2; TNF-α = Tumor Necrosis Factor-alpha; T-AOC = Total antioxidant capacity; SOD = Superoxide dismutase; GSH-Px = Glutathione peroxidase; CAT = Catalase; MDA = Malondialdehyde. ^a,b^ Means in the same row with no common letter differ significantly (*p* < 0.05).

**Table 6:** Dose-dependent effects of TBF on bone development and bone quality in Liangshan Yanying chickens.

Items	TBF, mg/kg				SEM	*p*-Value		
	0	20	40	60		ANOVA	Linear	Quadratic
Tibia Weight, g/kg	11.4	12.3	12.2	12.8	0.58	0.412	0.129	0.806
Tibia Length, cm	13.4	14.7	13.7	15	0.53	0.120	0.112	0.988
Tibia Diameter, mm	7.84	7.8	7.74	7.84	0.42	0.998	0.966	0.869
Tibia Plumpness, %	18.3	16.6	17.7	16.4	0.6	0.133	0.109	0.804
Tibia strength, kgf	20.9	21.9	21.3	23.2	0.91	0.334	0.137	0.606
Tibia ash content, %	39.9	43.5	42.9	42.7	0.96	0.071	0.086	0.064
Tibia calcium content, %	19.8	22	21	23.1	0.92	0.114	0.049	0.948
Tibia phosphorus content, %	8.39 ^b^	9.82 ^a^	9.06 ^ab^	9.48 ^a^	0.3	0.017	0.073	0.105
Femur Weight, %	8.95	9.9	9.82	10.6	1.31	0.784	0.346	0.946
Femur Length, cm	9.32	9.87	9.25	9.83	0.36	0.491	0.569	0.964
Femur Diameter, mm	7.99	8.31	8.44	8.53	0.36	0.736	0.291	0.762
Femur Plumpness, %	26.9	26.4	28.7	27.3	0.7	0.201	0.319	0.561
Femur strength, kgf	27.2	28.2	28.3	29.2	0.88	0.475	0.136	0.960
Femur ash content, %	48.3	49.5	51.7	50.1	0.85	0.070	0.060	0.112
Femur calcium content, %	22.3	22.4	22.7	24.6	0.78	0.170	0.059	0.255
Femur phosphorus content, %	10.1	10	10.3	10.2	0.25	0.829	0.545	0.961

^a,b^ Means in the same row with no common letter differ significantly (*p* < 0.05).

**Table 7:** Dose-dependent effects of TBF on carcass traits in Liangshan Yanying chickens.

Items	TBF, mg/kg				SEM	*p*-Value		
	0	20	40	60		ANOVA	Linear	Quadratic
Dressed percentage, %	82.7	84.4	81.5	83.6	0.82	0.097	0.981	0.807
Half-eviscerated percentage, %	75.4	74.7	74.8	74.6	0.65	0.805	0.408	0.672
Eviscerated percentage, %	64.5	64.8	64.2	65.1	0.6	0.767	0.654	0.632
Breast muscle percentage, %	18.2 ^b^	19 ^ab^	19.7 ^a^	19.7 ^a^	0.38	0.045	0.010	0.365
Thigh muscle percentage, %	23.4	24.7	24.2	24.4	0.63	0.520	0.353	0.438

^a,b^ Means in the same row with no common letter differ significantly (*p* < 0.05).

## Data Availability

The data presented in this study are available on request from the corresponding author due to the need to protect local chicken breed resources and related intellectual property rights.

## Abbreviations

The following abbreviations are used in this manuscript:

TBF	Tartary Buckwheat Flavonoids
BW	Body Weight
BWG	Body weight gain
FCR	Feed conversion ratio
ADFI	Average daily feed intake
ALB	Albumin
TC	Total cholesterol
LDL-C	Low-Density lipoprotein cholesterol
TG	Triglycerides
HDL-C	High-Density lipoprotein cholesterol
TP	Total protein
IgA	Immunoglobulin A
IgG	Immunoglobulin G
IgM	Immunoglobulin M
IL-2	Interleukin-2
TNF-α	Tumor Necrosis Factor a
T-AOC	Total antioxidant capacity
SOD	Superoxide dismutase
GSH-Px	Glutathione peroxidase
CAT	Catalase
MDA	Malondialdehyde
AMPKα1	AMP-activated protein kinase α1
SREBP-1C	Sterol regulatory element-binding protein-1C
ChREBP1	Carbohydrate response element-binding protein 1
ACC	Acetyl-CoA carboxylase
GPAT1	Glycerol-3-phosphate acyltransferase 1
FABP	Fatty acid-binding protein
FAS	Fatty acid synthase
PPARα	Peroxisome proliferator-activated receptor α
CPT1	Carnitine palmitoyltransferase 1

## References

[1] Wang T., Cui X., Li D., Chen B. (2024). Population genomics and transcriptomics identify patterns of selection in two Liangshan chicken breeds. Br. Poult. Sci..

[2] Oladokun S., Adewole D.I. (2020). In ovo delivery of bioactive substances: An alternative to the use of antibiotic growth promoters in poultry production—A review. J. Appl. Poult. Res..

[3] Chen S., Wang X., Cheng Y., Gao H., Chen X. (2023). A Review of Classification, Biosynthesis, Biological Activities and Potential Applications of Flavonoids. Molecules.

[4] Shen N., Wang T., Gan Q., Liu S., Wang L., Jin B. (2022). Plant flavonoids: Classification, distribution, biosynthesis, and antioxidant activity. Food Chem..

[5] Nagarajan S., Nagarajan R., Kumar J., Salemme A., Togna A.R., Saso L., Bruno F. (2020). Antioxidant Activity of Synthetic Polymers of Phenolic Compounds. Polymers.

[6] Li J., Dong J., Ouyang J., Cui J., Chen Y., Wang F., Wang J. (2017). Synthesis, characterization, solubilization, cytotoxicity and antioxidant activity of aminomethylated dihydroquercetin. Medchemcomm.

[7] Choy K.W., Murugan D., Leong X.F., Abas R., Alias A., Mustafa M.R. (2019). Flavonoids as Natural Anti-Inflammatory Agents Targeting Nuclear Factor-Kappa B (NFκB) Signaling in Cardiovascular Diseases: A Mini Review. Front. Pharmacol..

[8] Jiang F., Guan H., Liu D., Wu X., Fan M., Han J. (2017). Flavonoids from sea buckthorn inhibit the lipopolysaccharide-induced inflammatory response in RAW264.7 macrophages through the MAPK and NF-κB pathways. Food Funct..

[9] Yan Y., Xia X., Fatima A., Zhang L., Yuan G., Lian F., Wang Y. (2024). Antibacterial Activity and Mechanisms of Plant Flavonoids against Gram-Negative Bacteria Based on the Antibacterial Statistical Model. Pharmaceuticals.

[10] Tang E., Lin H., Yang Y., Xu J., Lin B., Yang Y., Huang Z., Wu X. (2024). Dietary astragalin confers protection against lipopolysaccharide-induced intestinal mucosal barrier damage through mitigating inflammation and modulating intestinal microbiota. Front. Nutr..

[11] Pavlova S.I. (2024). The role of microbiota and flavonoids in maintaining the balance of helper and regulatory T-lymphocytes associated with the intestinal immune barrier. Vopr. Pitan..

[12] Wang H., Huang B., Zhao W., Liu G., Bai W. (2026). Polyphenol-rich Chinese olive extracts attenuate lipid accumulation in HepG2 cells, accompanied by AMPK phosphorylation and miRNA alterations. Front. Nutr..

[13] Xiong X., Pangemanan J., Kiperman T., Sun Z., Paul A., Yechoor V., Ma K. (2025). Clock Modulation by Naringenin via RORα Suppresses Lipogenesis and Promotes Adipose Tissue Browning. bioRxiv.

[14] Tan Z., Halter B., Liu D., Gilbert E.R., Cline M.A. (2022). Dietary Flavonoids as Modulators of Lipid Metabolism in Poultry. Front. Physiol..

[15] Yang Y., Wei Z. (2025). Effects of total flavonoids from hawthorn leaves on growth performance, meat quality and economic benefits of broilers. China Feed.

[16] Kamboh A.A., Zhu W.Y. (2013). Effect of increasing levels of bioflavonoids in broiler feed on plasma anti-oxidative potential, lipid metabolites, and fatty acid composition of meat. Poult. Sci..

[17] Akter S., Rahman M.A., Siddique M.P., Hashem M.A., Chowdhury R. (2024). Use of a plant-based flavonoid blend in diet for growth, nutrient digestibility, gut microbiota, blood metabolites, and meat quality in broilers. J. Adv. Vet. Anim. Res..

[18] Jalal H., Doğan S.C., Giammarco M., Cavallini D., Lanzoni L., Pezzi P., Akram M.Z., Fusaro I. (2024). Evaluation of dietary supplementation of garlic powder (*Allium sativum*) on the growth performance, carcass traits and meat quality of Japanese quails (*Coturnix coturnix* japonica). Poult. Sci..

[19] Liu S., Fu S., Jin Y., Geng R., Li Y., Zhang Y., Liu J., Guo W. (2023). Tartary buckwheat flavonoids alleviates high-fat diet induced kidney fibrosis in mice by inhibiting MAPK and TGF-β1/Smad signaling pathway. Chem. Biol. Interact..

[20] Cui K., Wang Q., Wang S., Diao Q., Zhang N. (2019). The Facilitating Effect of Tartary Buckwheat Flavonoids and Lactobacillus plantarum on the Growth Performance, Nutrient Digestibility, Antioxidant Capacity, and Fecal Microbiota of Weaned Piglets. Animals.

[21] Ministry of Agriculture of the People’s Republic of China (2004). Chinese Feeding Standard for Chickens.

[22] Prihambodo T.R., Sholikin M.M., Qomariyah N., Jayanegara A., Batubara I., Utomo D.B., Nahrowi N. (2021). Effects of dietary flavonoids on performance, blood constituents, carcass composition and small intestinal morphology of broilers: A meta-analysis. Anim. Biosci..

[23] Paredes-Lopez D.M., Robles-Huaynate R.A., Perales-Camacho R.A., Alania-Santiago C.V., Diaz-Gonzales J.P., Aldava-Pardave U. (2025). Piper aduncum polyphenols and flavonoids enhance gut health, immune and anti-inflammatory activity and performance indices of broiler chickens. Front. Vet. Sci..

[24] Li A., Wang J., Wang Y., Zhang B., Chen Z., Zhu J., Wang X., Wang S. (2022). Tartary Buckwheat (*Fagopyrum tataricum*) Ameliorates Lipid Metabolism Disorders and Gut Microbiota Dysbiosis in High-Fat Diet-Fed Mice. Foods.

[25] Tan Y., Tam C.C., Rolston M., Alves P., Chen L., Meng S., Hong H., Chang S.K.C., Yokoyama W. (2021). Quercetin Ameliorates Insulin Resistance and Restores Gut Microbiome in Mice on High-Fat Diets. Antioxidants.

[26] Ma L., Lu Q.Y., Lim S., Han G., Boros L.G., Desai M., Yee J.K. (2025). The Effect of Flavonoids and Topiramate on Glucose Carbon Metabolism in a HepG2 Steatosis Cell Culture Model: A Stable Isotope Study. Nutrients.

[27] Shi L., Jin X., Xu Y., Xing Y., Yan S., Guo Y., Cheng Y., Shi B. (2022). Effects of Total Flavonoids of *Artemisia ordosica* on Growth Performance, Oxidative Stress, and Antioxidant Status of Lipopolysaccharide-Challenged Broilers. Antioxidants.

[28] Yuan J., Li Y., Miao J., Zhang X., Xiong Y., Ma F., Ding J., He S. (2025). Bamboo leaf flavonoids ameliorate cyclic heat stress-induced oxidative damage in broiler liver through activation of Keap1-Nrf2 signaling pathway. Poult. Sci..

[29] Goliomytis M., Tsoureki D., Simitzis P.E., Charismiadou M.A., Hager-Theodorides A.L., Deligeorgis S.G. (2014). The effects of quercetin dietary supplementation on broiler growth performance, meat quality, and oxidative stability. Poult. Sci..

[30] Xue F., Wan G., Xiao Y., Chen C., Qu M., Xu L. (2021). Growth performances, gastrointestinal epithelium and bacteria responses of Yellow-feathered chickens to kudzu-leaf flavonoids supplement. AMB Express.

[31] Tchana N.I., Wang S., Wang J., Nie W., Zhang S., Zhao Q., Tang C., Zhang J., Zhang H. (2026). Insight into the mechanism of Galega orientalis Lam. flavonoid extract in improving broiler meat antioxidative status and altering gut microbiota through network pharmacology. Poult. Sci..

[32] Tchana N.I., Zhang H., Pan Y., Wang S., Dansou D.M., Xia X., Zhao Q., Tang C., Zhang J. (2025). Dietary effect of Galega orientalis Lam. flavonoid extract on broilers’ growth performance, antioxidant capacity, immune function, and bone metabolism. Poult. Sci..

[33] Huang J., Tong X.F., Yu Z.W., Hu Y.P., Zhang L., Liu Y., Zhou Z.X. (2020). Dietary supplementation of total flavonoids from Rhizoma Drynariae improves bone health in older caged laying hens. Poult. Sci..

[34] Zhang F., Li Q., Wu J., Ruan H., Sun C., Zhu J., Song Q., Wei X., Shi Y., Zhu L. (2022). Total Flavonoids of Drynariae Rhizoma Improve Glucocorticoid-Induced Osteoporosis of Rats: UHPLC-MS-Based Qualitative Analysis, Network Pharmacology Strategy and Pharmacodynamic Validation. Front. Endocrinol..

[35] Shen Z., Chen Z., Li Z., Zhang Y., Jiang T., Lin H., Huang M., Chen H., Feng J., Jiang Z. (2020). Total Flavonoids of Rhizoma Drynariae Enhances Angiogenic-Osteogenic Coupling During Distraction Osteogenesis by Promoting Type H Vessel Formation Through PDGF-BB/PDGFR-β Instead of HIF-1α/VEGF Axis. Front. Pharmacol..

[36] Li L., Lu Z., Wang Y., Yang Y., Wang H., Ma H. (2024). Genistein alleviates chronic heat stress-induced lipid metabolism disorder and mitochondrial energetic dysfunction by activating the GPR30-AMPK-PGC-1α signaling pathways in the livers of broiler chickens. Poult. Sci..

[37] Liu Y., Sun Z., Dong R., Liu P., Zhang X., Li Y., Lai X., Cheong H.F., Wu Y., Wang Y. (2024). Rutin ameliorated lipid metabolism dysfunction of diabetic NAFLD via AMPK/SREBP1 pathway. Phytomedicine.

[38] Dai H., Lv Z., Huang Z., Ye N., Li S., Jiang J., Cheng Y., Shi F. (2021). Dietary hawthorn-leaves flavonoids improves ovarian function and liver lipid metabolism in aged breeder hens. Poult. Sci..

[39] Cao G., Wang H., Yu Y., Tao F., Yang H., Yang S., Qian Y., Li H., Yang C. (2023). Dietary bamboo leaf flavonoids improve quality and microstructure of broiler meat by changing untargeted metabolome. J. Anim. Sci. Biotechnol..

[40] Shen M.M., Zhang L.L., Chen Y.N., Zhang Y.Y., Han H.L., Niu Y., He J.T., Zhang Y.L., Cheng Y.F., Wang T. (2019). Effects of bamboo leaf extract on growth performance, meat quality, and meat oxidative stability in broiler chickens. Poult. Sci..

[41] Negm M.H., Aldhalmi A.K., Ashour E.A., Mohamed L.A., Kamal M., Rashad A., Khan M.M.H., Abd El-Hack M.E., Swelum A.A. (2025). Growth, Carcass Traits, Blood Chemistry and Gut Microbiota in Broiler Chickens Fed Diets Enriched With Garden Cress Seed Powder as a Natural Growth Enhancer. Vet. Med. Sci..

